# HIV-dependent depletion of influenza-specific memory B cells impacts B cell responsiveness to seasonal influenza immunisation

**DOI:** 10.1038/srep26478

**Published:** 2016-05-25

**Authors:** Adam K. Wheatley, Anne B. Kristensen, William N. Lay, Stephen J. Kent

**Affiliations:** 1 Department of Microbiology and Immunology at the Peter Doherty Institute for Infection and Immunity, The University of Melbourne, Melbourne, Australia; 2 ARC Centre of Excellence in Convergent Bio-Nano Science and Technology, University of Melbourne, Parkville, Australia; 3 Melbourne Sexual Health Centre and Department of Infectious Diseases, Alfred Health, Central Clinical School, Monash University, Melbourne, Australia

## Abstract

Infection with HIV drives significant alterations in B cell phenotype and function that can markedly influence antibody responses to immunisation. Anti-retroviral therapy (ART) can partially reverse many aspects of B cell dysregulation, however complete normalisation of vaccine responsiveness is not always observed. Here we examine the effects of underlying HIV infection upon humoral immunity to seasonal influenza vaccines. Serological and memory B cell responses were assessed in 26 HIV+ subjects receiving ART and 30 healthy controls immunised with the 2015 Southern Hemisphere trivalent inactivated influenza vaccine (IIV3). Frequencies and phenotypes of influenza hemagglutinin (HA)-specific B cells were assessed by flow cytometry using recombinant HA probes. Serum antibody was measured using hemagglutination inhibition assays. Serological responses to IIV3 were comparable between HIV+ and HIV− subjects. Likewise, the activation and expansion of memory B cell populations specific for vaccine-component influenza strains was observed in both cohorts, however peak frequencies were diminished in HIV+ subjects compared to uninfected controls. Lower circulating frequencies of memory B cells recognising vaccine-component and historical influenza strains were observed in HIV+ subjects at baseline, that were generally restored to levels comparable with HIV− controls post-vaccination. HIV infection is therefore associated with depletion of selected HA-specific memory B cell pools.

Infection with HIV leads to significant perturbations in B cell phenotype and function (reviewed in^1^) including polyclonal activation^2^,^3^,^4^, poor responsiveness to antigenic stimulation^4^,^5^ and a significant accumulation of normally minor populations of highly activated, anergic and/or exhausted B cells^4^,^6^,^7^. Immunologic and clinical manifestations include hypergammaglobulinemia^8^, a progressive depletion of CD27+ memory cells^9^,^10^,^11^ and diminished humoral responses to immunisation^10^,^12^,^13^,^14^. Initiation of anti-retroviral therapy (ART), particularly in the early phases of HIV infection, can partially reverse many aspects of B cell dysregulation^11^,^15^,^16^. However complete and sustained normalisation by ART is generally not observed^17^,^18^ suggesting that HIV infection leads to irreversible damage to the humoral immune system, or alternatively, aspects of HIV infection other than viremia sustain altered B cell phenotypes and functionality.

Many national health authorities recommend immunisation of HIV+ individuals against influenza due to potential for increased susceptibility and/or disease severity^19^. Conventional or adjuvanted influenza vaccines are immunogenic in HIV+ adults^20^,^21^,^22^,^23^ and children^24^,^25^,^26^,^27^, however the induction and maintenance of influenza-specific antibody titres is frequently lower than in comparable HIV− controls, particularly in the absence of ART or in infected subjects with low CD4 T cell counts^24^,^26^,^28^,^29^,^30^,^31^. Similarly, the establishment of influenza vaccine-elicited memory B cell responses, as measured by polyclonal stimulation and B cell ELISpot, are diminished compared to healthy controls^12^. Initiation of effective ART can restore serological responses to influenza vaccines to levels comparable with healthy controls^32^,^33^. However it remains unclear if dysfunction or depletion of antigen-specific memory B cell populations in ART-controlled infection affects vaccine-elicited immunity against influenza. The availability of recombinant, trimeric hemagglutinin flow cytometry probes^34^ facilitates the ready identification of HA-specific B cells directly *ex vivo* within clinical samples. Here we characterised influenza-specific antibody and memory B cell responses following administration of a seasonal inactivated trivalent influenza vaccine (IIV3) in HIV+ subjects receiving ART and healthy age-matched controls.

## Materials and Methods

### Ethics Statement

The study protocol was approved by both the Alfred Hospital Ethics Committee (#432/14), and the University of Melbourne Human Research Ethics Committee (#1443420) and all associated procedures were carried out in accordance with the approved guidelines. All participants provided written informed consent in accordance with the Declaration of Helsinki.

### Study Design and Clinical Samples

Trial design including detailed clinical characteristics of the subjects are fully described elsewhere^35^ and registered as NCT02632578 (http://www.clinicaltrials.gov/). Briefly, 27 HIV+ and 30 HIV− subjects (mean ages 41.4 and 40.4 years respectively) were vaccinated with the 2015 IIV3 (bioCSL Fluvax®) containing 15μg of hemagglutinin from A/California/7/2009-like (H1N1), A/Switzerland/9715293/2013 (H3N2)-like and B/Phuket/3073/2013-like strains. PBMCs were prepared and cryopreserved from blood samples taken prior to and ~4 weeks after vaccination. The current study utilised samples from all healthy controls and 26 of 27 recruited HIV+ subjects, who were on effective ART with a baseline plasma viral load of <100 HIV RNA copies/ml and a median CD4 count of 603/μl (interquartile range (IQR) 504–951). The HIV+ cohort had been infected for a median 6.1 years (IQR 2.5–12.2) and nadir CD4 counts were 310/μl (IQR 235–481).

### HA-specific B cell probes

Recombinant HA proteins for use as flow cytometry probes were derived for A/California/7/2009, A/Switzerland/9715293/2013, A/New Caledonia/20/1999 and A/Hong Kong/1/1968 strains as previously described^34^. Briefly, synthetic genes encompassing the ectodomain of HA modified to limit sialic acid binding were synthesised (GeneArt) and cloned into mammalian expression vectors. HA proteins were expressed by transient transfection of Expi293 (Life Technologies) suspension cultures and purified by polyhistidine-tag affinity chromatography and gel filtration. Proteins were biotinylated using BirA (Avidity) and stored at −80 °C. Prior to use, biotinylated HA proteins were labelled by the sequential addition of streptavidin (SA) conjugated to phycoerythrin (PE) or allophycocyanin (APC) and stored at 4 °C. A mock probe to control for specificity was generated using SA-BB515 (BD).

### Flow cytometry

HA-specific B cells were identified within a mean of 5.6 × 10^6^ cryopreserved PBMC (range 3.6–10.9 × 10^6^ PBMC/stain) by co-staining with HA probes conjugated to SA-PE or -APC (Thermofisher)^34^,^36^ and SA-BB515 probe control. Monoclonal antibodies for surface staining included: CD19-ECD (J3-119) (Beckman Coulter), IgM-BUV395 (G20-127), CD21-BUV737 (B-ly4), IgD-Cy7PE (IA6-2), IgG-BV786 (G18-145) (BD), IgA-Vio450 (IS11-8E10) (Miltenyi), CD14-BV510 (M5E2), CD3-BV510 (OKT3), CD8a-BV510 (RPA-T8), CD16-BV510 (3G8), CD10-BV510 (HI10a), CD27-BV605 (O323) (Biolegend). Cell viability was assessed using Aqua Live/Dead amine-reactive dye (Thermofisher). An average of 1.38 × 10^5^ live CD19+ B cells (interquartile range 0.86–1.83 × 10^5^) were collected per subject per time point (155 samples in total) using a BD Fortessa configured to detect 18 fluorochromes and analysis was performed using FlowJo software version 9.5.2 (TreeStar).

### Hemagglutination inhibition (HI) Assays

HI activity in serum samples was assessed using a standardised assay as previously described^37^, utilising 1% turkey erythrocytes (H1N1 subtypes) or 1% guinea pig erythrocytes (H3N2 subtypes). Briefly, serum samples were serially diluted in PBS from a starting dilution of 1:10 to 1:1280 prior to incubation with influenza viruses from strains B/Phuket/3073/2013, A/California/7/2009 or A/South Australia/55/2014 (an A/Switzerland/9715293/2013-like virus). HI titres are reported as the reciprocal of the highest dilution of serum where hemagglutination was completely inhibited.

### Statistical Analyses

Data is generally presented as median +/− interquartile range. Statistical significance was assessed by Mann-Whitney U tests. Correlations were analysed using Spearman’s rank-order tests. All statistical analyses were performed using Prism ver 5.0 (GraphPad).

## Results

### Baseline B cell characteristics in HIV+ and HIV− subjects

**Figure 1:**
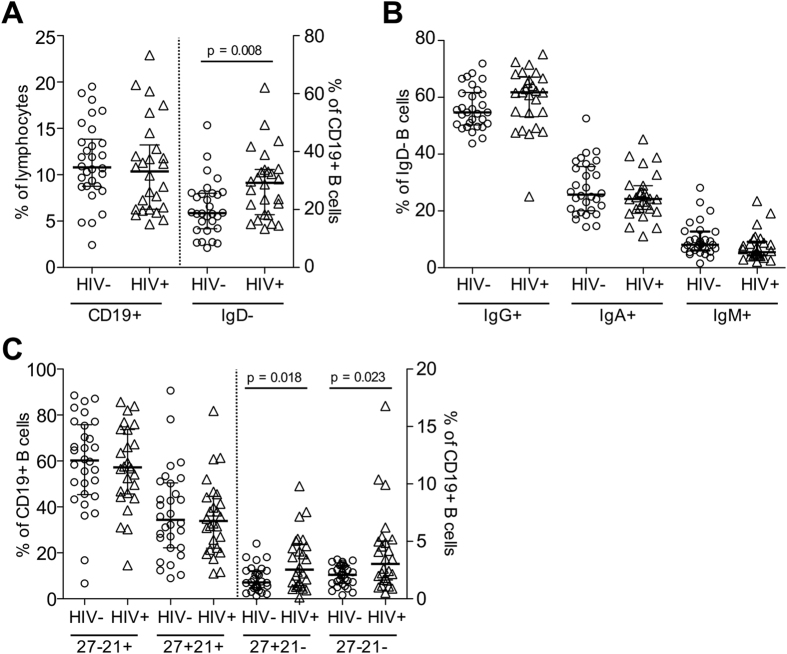
Baseline characteristics of B cells within PBMCs from HIV+ and HIV− subjects. (**A**) The frequencies of CD19+ lymphocytes and proportion of class-switched IgD- B cells was assessed by flow cytometry in HIV− (N = 30) and HIV+ subjects (N = 26). (**B**) Distribution of IgG, IgA and IgM expression memory B cells in both cohorts. (**C**) Distribution of CD19+ B cell subsets divided by CD27 and CD21 staining: CD27− CD21+ naïve, CD27+ CD21+ resting memory, CD27+ CD21− activated memory and CD27− CD21− tissue-like populations.

To examine any cohort specific differences associated with HIV infection, we first phenotypically characterised B cells at baseline in healthy volunteers (N = 30) and HIV+ individuals receiving ART (N = 26). An average of 5.6 × 10^6^ cryopreserved PBMCs were stained with monoclonal antibodies for surface markers including CD19, CD21, CD27, IgD, IgG, IgM and IgA (representative gating in Supplemental Fig. 1A) and analysed by flow cytometry. Pre-vaccination frequencies of CD19+ B cells were comparable between HIV− and HIV+ subjects at approximately 12% of lymphocytes, however an increased proportion of class-switched memory B cells (CD19+ IgD−) was observed in HIV+ subjects (28.85 ± 2.31%) compared with uninfected controls (20.55 ± 1.73%) (Fig. 1A). No changes were observed in the relative distributions of IgG, IgA and IgM subclasses of class-switched memory B cells (Fig. 1B). Differential staining for markers CD27 and CD21 can delineate distinct B cell subsets; CD27-CD21+ naïve B cells, CD27+CD21+ resting memory B cells, and two minor populations of CD27+CD21− activated memory and CD27-CD21− “tissue-like” memory B cells. The expansion of CD21− B cell subsets is a hallmark of chronic HIV infection^6^. Consistent with previous reports^16^, we observed elevated frequencies of both CD27+CD21− and CD27-CD21− B cell subsets in HIV+ subjects despite effective ART (Fig. 1C).

### Serum antibody responses to IIV3 immunisation

2015 saw the reformulation of the Southern Hemisphere IIV3 with a novel H3N2 component comprising an A/Switzerland/9715293/2013–like viral strain (SW13). Immunisation with the 2015 IIV3 therefore enables the de novo generation of immunity against an antigenically drifted influenza strain to be examined in the context of HIV infection. Serum antibody responses following IIV3 administration were measured in our cohorts using traditional hemagglutination inhibition assay (HI). Baseline HI activity against SW13 was observed in 13/30 and 15/25 of HIV− and HIV+ subjects tested respectively (Table 1). Following vaccination we observed a comparable rise in HI GMT against the H3N2 component in both HIV− and HIV+ subjects, with a respective 18/29 and 17/25 subjects displaying a >4-fold increases in GMT. In contrast, high baseline HI titres were observed to vaccine component H1N1 (A/California/7/2009) and B (B/Phuket/3073/2013) strains, with only low numbers of subjects showing >4-fold increases in GMT post-vaccination. Thus in terms of HI activity, the immunogenicity of the 2015 IIV3 vaccine was indistinguishable between HIV− and HIV+ cohorts, which may be reflective of the high CD4 counts and low viral loads associated with effective ART. A more detailed characterisation of the serological response to IIV3, in particular, differences in functional antibody responses between the cohorts are described elsewhere^35^.

### Memory B cell responses to H3N2 influenza

**Figure 2:**
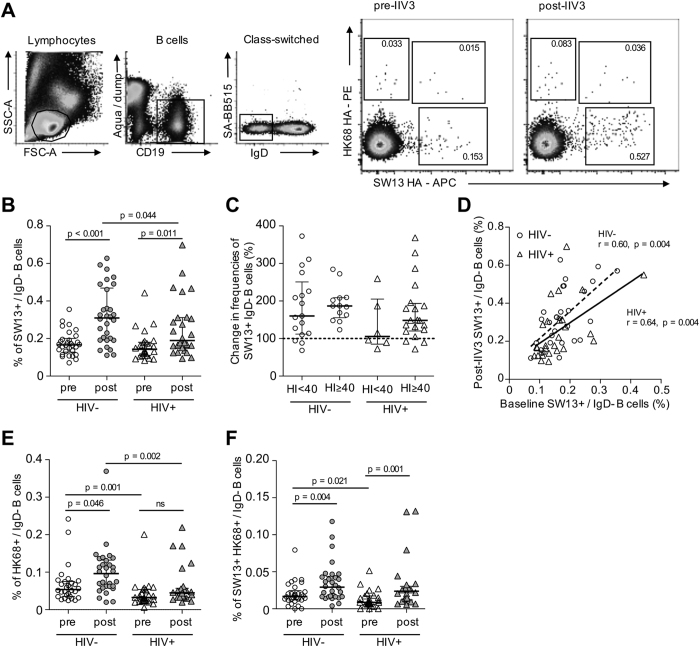
Memory B cell responses to H3N2 after IIV3 immunisation. (**A**) Cryopreserved PBMC samples were stained with a panel of monoclonal antibodies allowing identification of live CD19+IgD− B cells that did not bind an SA-BB515 mock probe control. Subsequent binding to A/Switzerland/9715293/2013 (SW13) and A/Hong Kong/1/1968 (HK68) probes allows the identification of three distinct populations of IgD− memory B cells; SW13+, HK68+ and a cross-reactive SW13+HK68+ population. Shown for a representative HIV− individual. (**B**) Frequencies of SW13+ memory B cells prior to and following IIV3 vaccination in HIV+ (N = 26) and HIV− subjects (N = 30). (**C**) Degree of SW13+ memory B cell expansion (%) in HIV+ and HIV− subjects segregated by baseline seropositivity (HI ≥ 40) to a SW13-like virus as measured by HI assay. (**D**) Correlation between SW13+ memory B cell frequencies at baseline and post-IIV3. (**E**) HK68+ and (**F**) cross-reactive SW13+HK68+ memory B cell frequencies before and following IIV3. Data is shown as median+/−interquartile range. Populations were compared using Mann-Whitney U tests and p values denoted. Correlations were assessed using Spearman’s rank-order test and r and p values are denoted. ns; not significant.

Given the previous literature on HIV-associated B cell dysregulation^1^ we next characterised memory B cell responses to H3N2 influenza. Recombinant HA probes were derived as previously described^34^ for both SW13 and an historical H3N2 strain A/Hong Kong/1/1968 (HK68). Class-switched IgD- memory B cells from HIV+ and HIV− subjects were co-stained with H3 probes. Three populations of HA-binding memory B cells could be delineated: SW13+, HK68+ and SW13+HK68+ cross-reactive B cells (gating and representative staining in Fig. 2A). Despite A/Switzerland/9715293/2013 being a very recently circulating strain, SW13+ memory B cells were detectable in all baseline samples in both HIV− and HIV+ subjects at frequencies of 0.17 ± 0.01% and 0.16 ± 0.02% of the IgD- population respectively (Fig. 2B). Following IIV3 vaccination, we observed expansion of SW13+ memory B cells in both cohorts, although post-vaccination frequencies were significantly higher in HIV− individuals. Interestingly, when stratified on the basis of baseline seropositivity (defined as HI ≥ 40), we found diminished expansion of SW13+ memory B cells was primarily concentrated in HIV+ subjects seronegative at baseline (Fig. 2C). However due to the small number of individuals examined, this observation requires confirmation in larger cohorts. In both HIV+ and HIV− subjects, SW13+ B cell frequencies at baseline were proportional to those observed post-IIV3 (Fig. 2D). Moreover, we observed a weak but significant correlation (r = 0.299, p = 0.028) between the magnitude of IIV3-induced expansion in SW13+ memory B cell frequencies and the increase in serum HI titres (Supplementary Fig. 2A), however the degree of inter-relationship between HA-specific B cells and serological neutralising antibody responses is currently unclear.

At baseline, memory B cells recognising HK68 alone (Fig. 2E) or SW13+HK68+ cross-reactive cells (Fig. 2F) were comparatively infrequent and present at higher frequencies in HIV− versus HIV+ subjects. This observation suggests HIV infection may be associated with the depletion of memory B cell pools recognising historical H3N2 strains (HK68) and those recognising highly cross-reactive epitopes conserved in currently circulating H3N2 strains (SW13+HK68+). In response to vaccination, both HK68+ and SW13+HK68+ memory B cells expanded in HIV− subjects, while only SW13+HK68+ memory B cells underwent expansion in HIV+ subjects. Historical drift within H3N2 isolates would suggest that A/Hong Kong/1/1968 and contemporary strains like SW13 bridge considerable antigenic distance. Therefore the expansion of HK68+ memory B cells by a heterologous IIV3 vaccine is somewhat unexpected, but may reflect the expansion of rare B cell lineages cross-reactive to H1N1 or influenza B components of the trivalent vaccine^38^,^39^. We observed no correlation between post-IIV expansions of HK68+ and SW13+HK68+ cross-reactive B cells (Supplementary Fig. 3).

### Phenotype of SW13+ memory B cells

**Figure 3:**
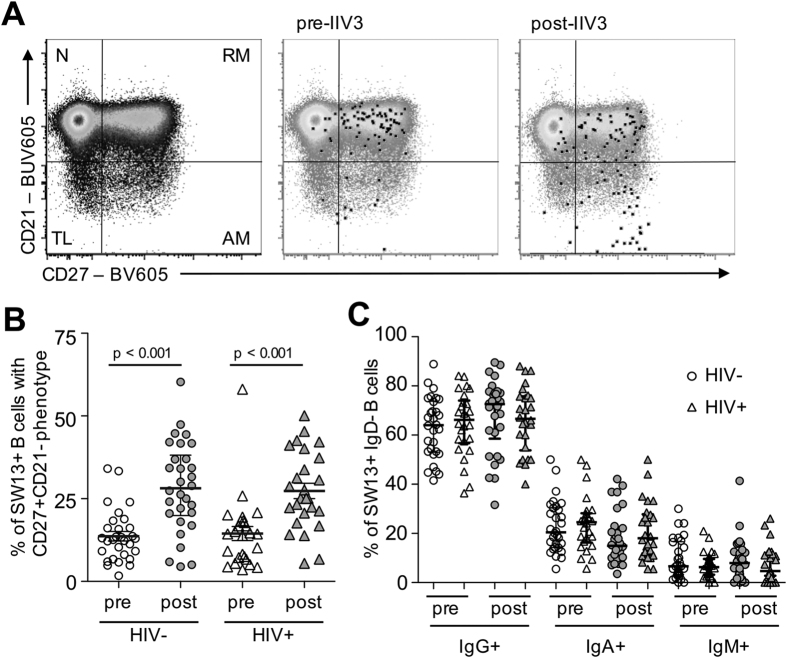
Phenotype of SW13+ memory B cells. (**A**) Staining of surface markers CD21 and CD27 is shown for back-gated SW13+ memory B cells relative to the parental CD19+ population before or after vaccination. CD27− CD21+ naïve (N), CD27+ CD21+ resting memory (RM), CD27+ CD21− activated memory (AM) and CD27− CD21− tissue-like populations (TL) are denoted. (**B**) The proportion of SW13+ memory B cells with an activated memory (CD27+CD21−) phenotype before and after IIV3 immunisation in HIV+ (N = 26) and HIV− subjects (N = 30). (**C**) Proportions of IgG, IgA and IgM immunoglobulin subclass expression upon SW13+ memory B cells before and after immunisation.

A major advantage the pairing of HA probes and flow cytometry has over traditional ELIspot based protocols for enumerating memory B cells is the ability to simultaneously phenotype antigen-specific memory B cells based on surface marker expression. We next characterised the phenotype of SW13+ memory B cells. Down-regulation of CD21 (complement receptor-2; CR2) is a hallmark of B cell activation and differentiation^40^,^41^. Migration of SW13+ B cells from a resting memory phenotype (CD27+CD21+) to an activated phenotype (CD27+CD21−) was observed following IIV3 immunisation (Fig. 3A). Elevated proportions of activated SW13+ memory B cells were seen in both HIV− and HIV+ subjects (Fig. 3B), consistent with phenotypic changes previously described for memory B cells responsive to H5N1 vaccination^36^. Vaccination did not however change the relative distributions of IgG-, IgA- and IgM-expressing memory subsets among SW13+ B cells (Fig. 3C), which largely mirrored the proportions seen for the parental IgD- B cell population.

### Memory B cell responses to H1N1 influenza

**Figure 4:**
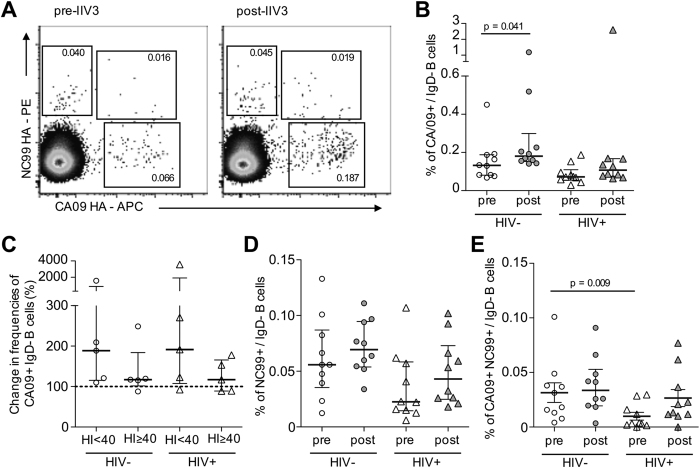
(**A**) Memory B cell responses to H1N1. PBMC samples were co-stained as before with HA probes for H1N1 strains A/California/4/2009 (CA09) and A/New Caledonia/20/1999 (NC99). (**B**) Frequencies CA09+ memory B cells before and following IIV3 vaccination in HIV+ (N = 10) and HIV− subjects (N = 10). (**C**) Degree of CA09+ memory B cell expansion in HIV+ and HIV− subjects segregated by baseline HI activity seropositivity (HI ≥ 40) to CA09. (**D**) NC99+ and (**E**) cross-reactive CA09+NC99+ memory B cell frequencies before and following IIV3. Data is shown as median +/− interquartile range. Populations were compared using Mann-Whitney U tests and p values denoted.

We next examined memory B cell responses in our cohorts to the H1N1 component (A/California/7/2009; CA09) of the 2015 IIV3. Due to limited clinical sample availability, we limited these analyses to a smaller subset of vaccine recipients. CA09 has been circulating for 6 years and baseline seropositivity (defined as HI ≥ 40) was widespread prior to IIV3 administration (Table 1; 20/30 of HIV−, 18/25 of HIV+ subjects). Given our observations with H3N2 that baseline antibody responses may influence subsequent memory B cell responsiveness, H1N1-specific memory B cells were characterised before and after IIV3 in HIV− (n = 10; 5 seropositive at baseline and 5 negative) and HIV+ (n = 10; 5 seropositive at baseline and 5 negative) individuals. HA probes were derived from CA09 and an historical heterologous H1N1 virus A/New Caledonia/20/1999 (NC99) and PBMC samples stained as before (representative staining in Fig. 4A). The degree of memory B cell expansion was more variable than that observed for the H3 B cell responses despite the smaller sample size. However a significant expansion of CA09+ memory B cells was observed in HIV− but not HIV+ subjects (Fig. 4B). Although most subjects showed modest increases in post-vaccination CA09+ B cell frequencies, up to 20–40 fold increases were observed in selected individuals. When divided based upon pre-existing HI activity to CA09, the magnitude of CA09+ B cell expansion was comparatively diminished in individuals seropositive at baseline (Fig. 4C). Analogous with SW13 responses, the magnitude of IIV3-induced expansion in CA09+ memory B cell frequencies correlated with the degree to which serum HI titres increased post-immunisation (r = 0.748, p < 0.001; Supplementary Fig. 2B).

Analogous to observations with a heterologous H3N2 strain, we observed a trend toward decreased NC99+ (Fig. 4D) and a significant reduction in H1N1 cross-reactive memory B cell frequencies (Fig. 4E) in HIV+ subjects at baseline. However IIV3 administration led to expansion of NC99+ and CA09+NC99+ B cells in most individuals, with circulating frequencies restored to levels comparable to HIV− subjects.

## Discussion

There is a need to better understand B cell responses to IIV3 vaccines, the mainstay of influenza prevention. This is particularly true when antigenic drift diminishes efficacy and forces vaccine reformulation (such as the introduction of A/Switzerland/9715293/2013 in 2015). There is also a need to understand B cell responses to IIV3 vaccines in subjects with compromised immunity since they are at the greatest risk of severe disease. We found the 2015 IIV3 induced serological responses generally comparable between HIV− and HIV+ subjects on ART as measured by traditional HI assays, consistent with past reports demonstrating ART restores effective antibody responses to influenza^32^ and other viral vaccines^42^. However serological studies based on HI titres alone constitute a constricted overview of humoral immunity to influenza vaccination. We recently described diminished influenza-specific antibody responses with Fc-functions, such as antibody-dependent cellular cytotoxicity and antibody-dependent phagocytosis, were evident in baseline serum samples from HIV+ individuals^35^. However, IIV3 immunisation drove the restoration of these responses to levels equivalent in uninfected controls. There is a also need to more accurately enumerate and phenotype B cells responding to seasonal influenza vaccines. Here we utilised novel HA probes to characterise HA-specific memory populations responding to IIV3 directly *ex vivo*. IIV3-induced memory B cell expansion and phenotypic changes consistent with activation was observed in both HIV− and HIV+ individuals, however lower post-vaccination frequencies of both H3N2 and H1N1 specific memory B cells were seen in HIV+ subjects. Impaired induction of memory B cells by influenza vaccines has been reported in the context of viremic infection^12^. However, our results contrast with previous findings that memory B cell expansion was comparable between HIV+ subjects on ART and healthy controls following seasonal influenza vaccination^47^. The enumeration of memory B cells has traditionally utilised polyclonal stimulation and the subsequent detection of secreted immunoglobulin by B cell ELISPOT. However staining B cells directly with HA probes allows direct enumeration via binding to surface immunoglobulin, thereby decoupling memory B cell proliferative potential from measurements of antigen specificity. This may be important in the context of HIV infection where B cell dysregulation is evident. However, it is unclear if differences between our study and others are cohort specific or reflective of alternative methodologies used for memory B cell enumeration. Future investigations utilising expanded cohorts are needed to clarify how methodology, complicated immune landscapes established by prior influenza exposure, subject age and specific vaccine formulations combine to influence memory B cell responsiveness to seasonal influenza vaccines.

We found at least two distinct factors influenced elicitation of influenza-specific memory B cell responses following administration of IIV3. Firstly, the magnitude of memory B cells induced post-vaccination was proportional to initial frequencies observed at baseline. And secondly, pre-existing serological responses can influence the magnitude of memory B cell expansion after immunisation. We found CA09+ memory B cell responses were diminished in subjects with baseline HI activity, supporting previous studies showing pre-existing HI activity negatively correlates with plasmablast expansion and the development of serum antibody responses following seasonal influenza vaccination^48^,^49^. However this did not seem to be the case with regards to the H3N2 vaccine component, where pre-existing HI activity did not reduce subsequent memory B cell expansion to IIV3. It is unclear to what extent these differences are strain-specific. SW13-specific HI activity at baseline presumably reflects cross-reactive antibody elicited by historical exposure to antigenic drift variants of H3N2. In contrast, baseline HI activity to the recently emerged pandemic H1N1 (such as CA09), which has undergone limited antigenic drift to date, likely reflects direct infection or previous vaccination against CA09 or matched strains. Qualitative differences in the antibody response to CA09 and SW13 likely contribute to the differential effect seen with regards to suppression of homologous memory B cell elicitation by IIV3.

Baseline seropositivity rates for H1N1 and H3N2 strains included in the 2015 IIV3 were comparable between HIV+ and HIV− subjects. Interestingly, significant populations of memory B cells recognising SW13 were observed in all subjects at baseline. This observation suggests repeated lifetime exposures to historical H3N2 antigens seeds memory B cells with cross-reactivity to as yet un-encountered H3N2 strains which have undergone antigenic drift. In the context of HIV infection, baseline frequencies of memory B cells to either vaccine homologous, or heterologous influenza strains, were consistently lower in HIV+ subjects, reaching statistical significance in many cases. Similarly, memory B cells highly cross-reactive between H3N2, or between H1N1 seasonal strains, were present at lower frequencies in HIV+ subjects. The mechanistic basis underpinning decreased memory B cell frequencies remains to be clarified. It is possible that non-specific polyclonal activation during acute and/or viremic infection prior to commencement of ART may lead to a purging of influenza-specific memory B cells elicited by historical exposure, analogous to reports for other pathogens^10^,^50^. Supporting this idea, memory B cell depletion following Rituximab treatment in lymphoma patients leads to impaired humoral responses to IIV3^51^. Alternatively, persistent B cell dysregulation despite effective ART, evidenced in the current study by elevated frequencies of CD21− B cell subsets, may be indicative of other underlying defects in the humoral compartment leading to impaired maintenance or generation of B cell memory.

The immunological consequences of diminished frequencies of influenza-specific memory B cells with regards to influenza infection or disease are unclear. However depletion of memory B cells following measles infection was recently linked to long-term immunosuppression and increased susceptibility to infection^52^. HA-specific memory B cells have the capacity to respond rapidly to recall antigen during secondary influenza exposure^53^. Furthermore in adult humans, B cells recruited into humoral responses to antigenically novel strains are thought to be drawn predominantly from the memory pool^54^,^55^. Notably, B cells binding highly cross-reactive epitopes, such as those presumably recognised by CA09+NC99+ or SW13+HK68+ memory B cells in the current study, are enriched for heterosubtypic neutralising specificities such as those binding the HA stem^36^,^55^,^56^. Therefore it remains a distinct possibility that selective depletion of influenza-specific memory B cells and in particular, highly cross-reactive sub-populations, may increase susceptibility to influenza infection or disease in HIV infected subjects. This may be of particular importance during periodic influenza pandemics with antigenic shift variants such as the emergent pdmH1N1 virus in 2009, where pre-existing cross-neutralising humoral immunity may lower susceptibility and/or disease severity^57^,^58^.

Our results suggest that annual IIV3 vaccination of HIV-infected individuals is important to maintain or re-establish pools of B cell memory. Improved vaccination strategies, such as the incorporation of novel adjuvants^23^,^59^,^60^,^61^, may be required to efficiently repopulate influenza-specific B cell populations in this vulnerable group. Further investigations into the mechanistic basis and clinical consequences of influenza-specific memory B cell depletion in the context of HIV infection should be explored.

## Additional Information

**How to cite this article**: Wheatley, A. K. *et al*. HIV-dependent depletion of influenza-specific memory B cells impacts B cell responsiveness to seasonal influenza immunisation. *Sci. Rep.* **6**, 26478; doi: 10.1038/srep26478 (2016).

## Supplementary Material

Supplementary Information

## Figures and Tables

**Table 1:** Serum hemagglutination inhibition titres before and after IIV3 in HIV− subjects (baseline: N = 30, post-IIV3: N = 29) and HIV+ subjects (baseline: N = 25, post-IIV3: N = 26).

	HIV negative		HIV positive	
	Baseline	Post-IIV3	Baseline	Post-IIV3
H3N2 - A/South Australia/55/2014 (A/Switzerland/9715293/2013-like)				
Seropositive (HI ≥ 40)	13 (43%)	25 (86%)	15 (60%)	24 (92%)
GMT	28.3	126.0	29.5	140.0
≥4-fold rise post-IIV3	18/29 (62%)		17/25 (68%)	
H1N1 - A/California/7/2009				
Seropositive (HI ≥ 40)	20 (67%)	25 (86%)	18 (72%)	24 (92%)
GMT	40.9	66.1	48.6	93.9
≥4-fold rise post-IIV3	3/29 (10%)		5/25 (20%)	
B Yamagata- B/Phuket/3073/2013				
Seropositive (HI ≥ 40)	24 (80%)	27 (93%)	17 (68%)	22 (85%)
GMT	81.9	156.2	49.9	104.4
≥4-fold rise post-IIV3	5/29 (17%)		8/25 (32%)	

GMT; geometric mean titre.
