# Supplementary material for: HIV-dependent depletion of influenza-specific memory B cells impacts B cell responsiveness to seasonal influenza immunisation
Source: Sci Rep. 2016 May 25;6:26478. doi: 10.1038/srep26478 (PMC4879526; doi:10.1038/srep26478)
Supplement: Supplementary Information [file srep26478-s1.pdf]

HIV-dependent depletion of influenza-specific memory B cells impacts B cell responsiveness to seasonal influenza immunisation

Adam K Wheatley*[1,3], Anne B Kristensen[1], William N Lay[1] and Stephen J Kent[1,2,3]

[1] Department of Microbiology and Immunology at the Peter Doherty Institute for Infection and Immunity, The University of Melbourne, Melbourne, Australia
[2] Melbourne Sexual Health Centre and Department of Infectious Diseases, Alfred Health, Central Clinical School, Monash University, Melbourne, Australia
[3] ARC Centre of Excellence in Convergent Bio-Nano Science and Technology, University of Melbourne, Parkville, Australia

***Corresponding Author**: AK Wheatley, Department of Microbiology and Immunology, University of Melbourne at the Peter Doherty Institute for Infection and Immunity, 792 Elizabeth Street, Melbourne VIC 3000, Australia.
Tel.: +61390354179; fax: +61 383445740;
E-mail address: a.wheatley@unimelb.edu.au

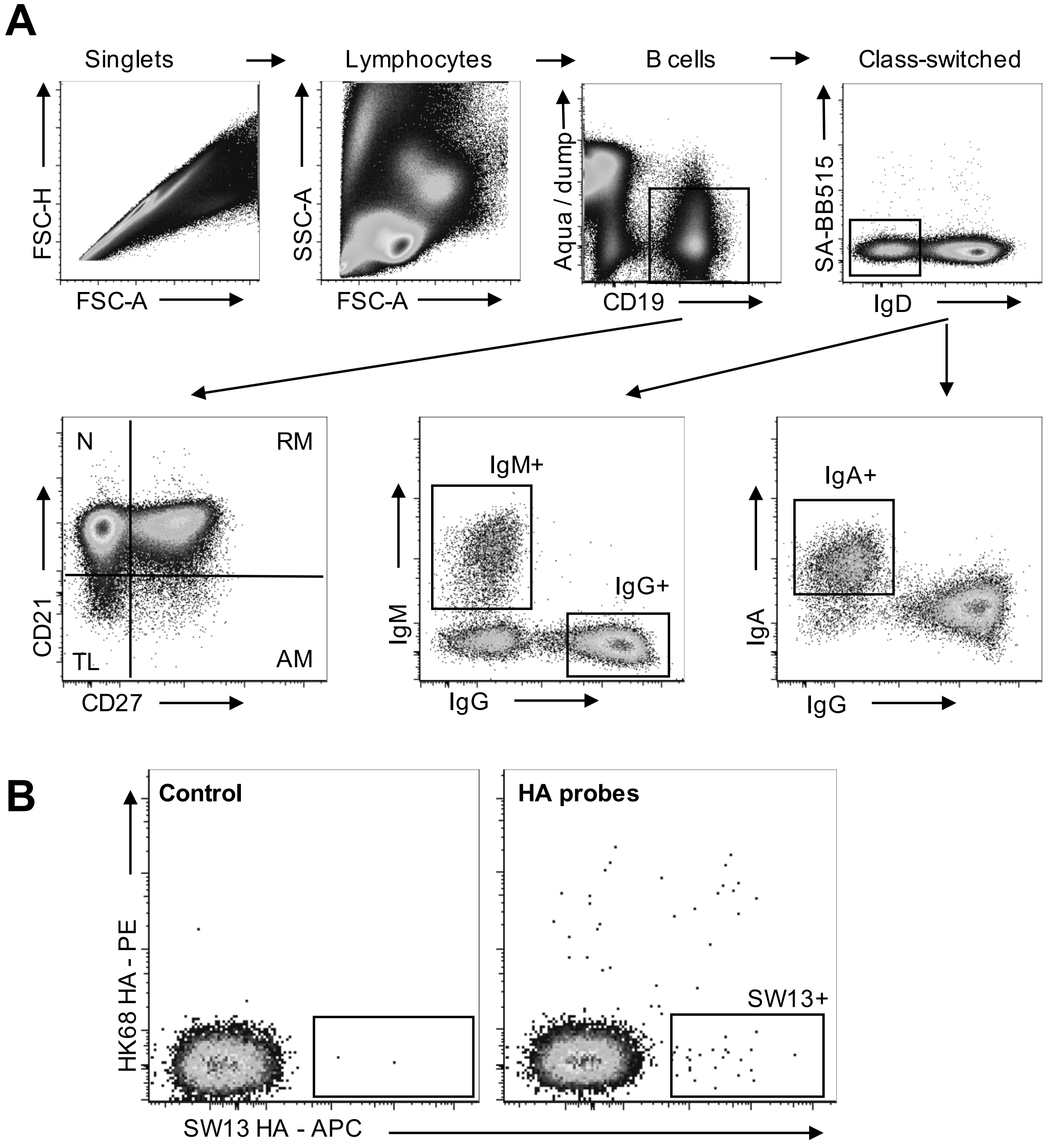

Supplementary Figure 1. Representative flow cytometry gating. (**A**) The phenotype of single, live CD19+ B lymphocytes was assessed using surface markers CD21 and CD27. CD27- CD21+ naïve (N), CD27+ CD21+ resting memory (RM), CD27+ CD21- activated memory (AM) and CD27- CD21- tissue-like populations (TL) are denoted. Surface immunoglobulin expression was determined by co-staining for IgG, IgA and IgM subclasses. (**B**) Class-switched B cells not binding decoy streptavidin-BB515 (SA-BB515) were co-stained with recombinant HA probes. Positive gates were established with reference to cells stained with control probes (SA-conjugates alone).

A

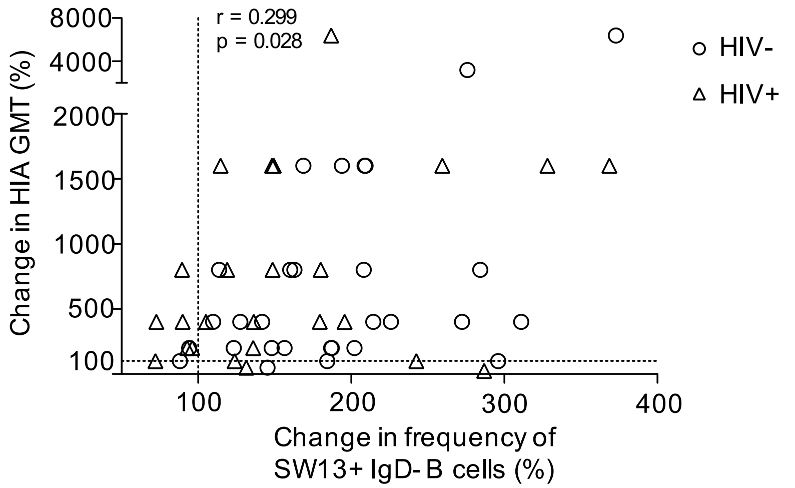

B

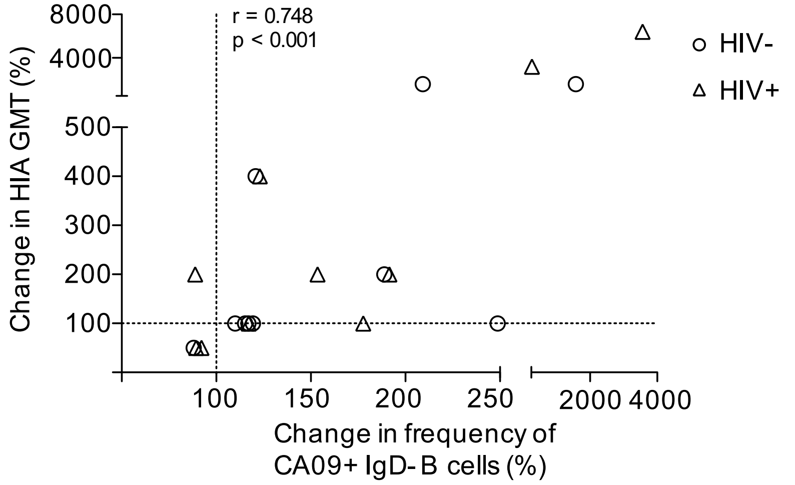

Supplementary Figure 2. Correlations between IIV3-elicited changes in (**A**) SW13+ or **(B)** CA09+ memory B cell frequencies and HI activity (GMT).

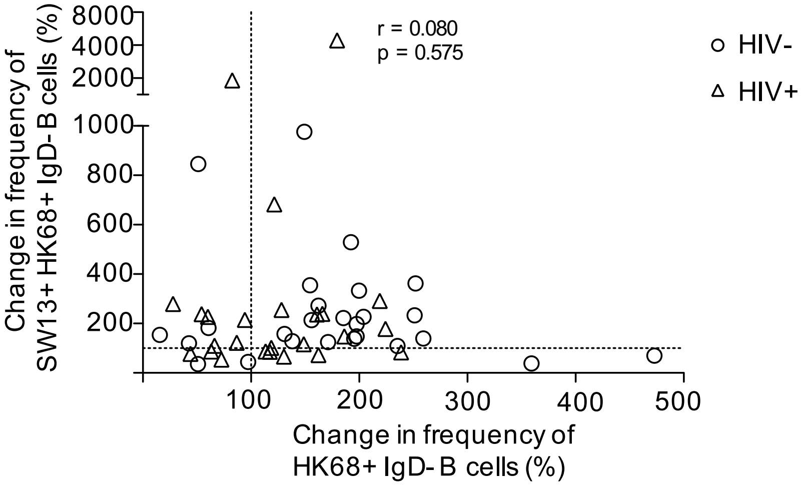

Supplementary Figure 3. No correlation between post-IIV3 expansions of HK68+ and HK68+SW13+ memory B cells.
